# Opportunities and challenges of immunotherapy for dMMR/MSI-H colorectal cancer

**DOI:** 10.20892/j.issn.2095-3941.2023.0240

**Published:** 2023-10-20

**Authors:** Qi Zhang, Jian Li, Lin Shen, Yongsheng Li, Xicheng Wang

**Affiliations:** 1 Department of Medical Oncology, Chongqing University Cancer Hospital, Chongqing 400030, China; 2 Department of Gastrointestinal Oncology, Laboratory of Carcinogenesis and Translational Research of the Ministry of Education, Peking University School of Oncology, Beijing Cancer Hospital & Institute, Beijing 100142, China

Colorectal cancer (CRC) with mismatch-repair deficiency (dMMR) is a distinct molecular subgroup of tumors that is characterized by a diminished capacity to correct base-pair mismatches in DNA, which leads to changes in microsatellite sequences and results in high microsatellite instability (MSI-H) accompanied by hypermutation^1^. The incidence of dMMR/MSI-H in patients with CRC has been reported to be 15% (12% with sporadic disease and 3% with Lynch syndrome). The incidence varies by stage, with 20% in stage II, 10%–15% in stage III, and 3%–5% in stage IV^2^. Despite the presence of numerous neoantigens that promote tumor-infiltrating lymphocytes in the microenvironment, dMMR/MSI-H CRC also exhibits significantly increased expression of immune checkpoints, such as PD-1 and CTLA-4^3^. Immune checkpoint inhibitor (ICI) therapy has been shown to have high sensitivity for dMMR/MSI-H metastatic CRC (mCRC). Additionally, promising results have been obtained in several trials involving locally advanced dMMR/MSI-H CRC. As the search of ICIs for dMMR/MSI-H CRC treatment continues, clinical practice faces numerous opportunities and challenges (**Figure 1**).

**Figure 1:**
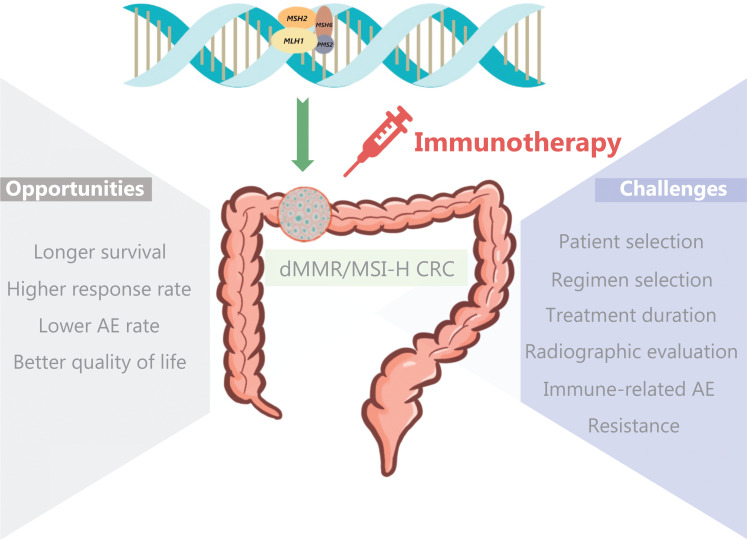
Summary of opportunities and challenges of immunotherapy for dMMR/MSI-H colorectal cancer. AE, adverse event; dMMR, mismatch-repair deficiency; MSI-H, high microsatellite instability; CRC, Colorectal cancer.

## ICIs for dMMR/MSI-H mCRC

Since achieving a 3-year complete response (CR) in the first pretreated MSI-H CRC patient with anti-PD-1 antibody treatment^4^, several studies have been conducted to investigate the use of ICI in pretreated dMMR/MSI-H mCRC patients (**Table 1**). The results have shown that the objective response rate (ORR) ranges from 32%–63% with a disease control rate (DCR) of 51%–82% when dMMR/MSI-H CRC patients are treated with a single-agent ICI. Additionally, the 1-year progression-free survival (PFS) and overall survival (OS) rates were 34%–63% and 72%–87%, respectively. Notably, the combination of nivolumab and ipilimumab yielded a more promising response with an ORR of 65% and a DCR of 81%, which indicates a long-term survival benefit^7^.

**Table 1:** Efficacy of immune checkpoint inhibitor treatment of MSI-H/dMMR colorectal cancer in select clinical trials

Trial	KEYNOTE 164^5^		KEYNOTE 016^6^	CheckMate 142^7,8^		Li et al.^9^	Li et al.^10^		Astrum010^11^
Prior treatment line	≥ 1	≥ 2	≥ 2	≥ 1		≥ 2	1	≥ 2	≥ 2
Treatment	Pembrolizumab		Pembrolizumab	Nivolumab	Nivolumab +Ipilimumab	Tislelizumab	Envafolimab	Envafolimab	Serplulimab
Sample size (*n*)	63	61	40	74	119	46	24	41	53
Follow-up time	24	31	/	21	51	15	12		/
cCR (%)	8	3	13	3	13	4	13	0	/
ORR (%)	33	33	52	34	65	39	63	32	38
DCR (%)	57	51	82	62	96	72	67	59	/
DoR (month)	4.4 to 23.6+	6.2 to 31.3+	/	1.4 to 31.6	1.4+ to 50.8+	NR	2.8+ to 15.0+	1.0+ to 16.6+	1.6 to 13.1
PFS (month)	4.1	2.3	NR	6.6	NR	NR	NR	4.9	/
1Y-PFS (%)	41	34	/	44	71	/	63	32	/
2Y-PFS (%)	37	31	59	NR	60	/	/	/	/
1Y-OS (%)	76	72	/	72	85	/	87	65	/
2Y-OS (%)	63	55	72	/	74	/	/	/	/

cCR, clinical complete response; DCR, disease control rate; DoR, duration of response; NR, not reported; ORR, objective response rate; OS, overall survival; PFS, progression-free survival; Y, year.

In the first randomized phase III trial for dMMR/MSI-H mCRC, KEYNOTE 177 demonstrated that single-agent anti-PD-1 therapy was more effective than chemotherapy as the initial treatment option. The pembrolizumab group (*n* = 153) had a PFS of 16.5 months, whereas the chemotherapy group (*n* = 154) had a PFS of 8.2 months. The ORR was 43.8%, and 11.1% of patients in the pembrolizumab group achieved a clinical CR (cCR)^12^. Moreover, in the subgroup of patients receiving first-line treatment in the phase II Checkmate 142 trial (*n* = 45), the combination of nivolumab and ipilimumab demonstrated promising results^13^. The ORR was 69% and the DCR was 89%. Additionally, the 2-year PFS and OS rates were 73.6% and 79.4%, respectively^13^.

### Challenge of optimal ICI selection for dMMR/MSI-H mCRC in first-line therapy

Dual ICIs that target different immune checkpoints may be a more effective approach, as suggested by the CheckMate 142 findings^7,13^. It is noteworthy that using a single-agent ICI did not have a significant impact on patients with a *RAS* mutation^12^. Dual ICIs, however, showed benefits for patients regardless of *KRAS* and *BRAF* status^7,13^. Indeed, it is important to consider the economic cost and adverse events (AEs) associated with dual ICIs. Additionally, it has been observed that single-agent immunotherapy is sufficient for some patients^12^. Currently, the ongoing CheckMate 8HW trial (NCT04008030) is comparing the efficacy of nivolumab alone, nivolumab plus ipilimumab, and chemotherapy in dMMR/MSI-H mCRC patients, which may provide valuable insight for selecting between single-agent and dual immunotherapy. Another promising approach is the combination of ICI and targeted therapy, particularly anti-angiogenesis, which can transform the tumor microenvironment from immune-suppressive to immune-supportive. We eagerly await the results of the COMMIT trial (NCT02997228), which is evaluating dMMR/MSI-H CRC first-line treatment using atezolizumab, chemotherapy, and bevacizumab.

### Challenge of ICI resistance in dMMR/MSI-H mCRC

Despite the promising results, > 50% of patients experience resistance to single-agent ICIs^12^. The molecular mechanism underlying ICI resistance in dMMR/MSI-H CRC has not been elucidated. While *RAS* mutations in CRC have been extensively studied, the impact on effectiveness of immunotherapy in dMMR/MSI-H CRC is not completely understood. In pretreated patients with *RAS* mutations, the response rate to single-agent ICI is approximately 35%, which is significantly higher than the response rate to chemotherapy (6.7%)^5,14^. Patients with *RAS* mutations did not realize a PFS benefit with single-agent ICI compared to chemotherapy in the KEYNOTE 177 trial^12^. A retrospective analysis revealed that MSI-H CRC with a *RAS* mutation had reduced immunogenicity, characterized by upregulated WNT/SHH pathway activity and a decreased neoantigen tumor burden^15^. In contrast, MSI-H CRC with a *BRAF V600E* mutation had a similar neoantigen tumor burden and extent of T-cell infiltration compared to MSI-H CRC with wild-type *BRAF*^16^. Interestingly, patients with a *BRAF V600E* mutation had a better PFS with single-agent ICI compared to chemotherapy^12,13^.

Chida and colleagues^17^ reported that compared to wild-type *PTEN*, a *PTEN* mutation, particularly in the phosphatase domain, led to a significantly lower efficacy of ICI treatment (ORR, 12.5% *vs.* 54.8%; PFS, 2.6 months *vs.* 15.6 months; OS, 15.2 months *vs.* 25.7 months) in dMMR/MSI-H gastrointestinal (GI) cancer patients. This difference in efficacy was attributed to a more immunosuppressive tumor environment^17^. Furthermore, another study identified *AKT1* and *CDH1* mutations as potential biomarkers for primary resistance to ICIs in dMMR/MSI-H GI cancer^18^. Patients with *AKT1* or *CDH1* mutations exhibited a significantly worse prognosis in both the discovery (*n* = 65: PFS, 2.1 months *vs.* NR, *P* < 0.001; OS, 16.9 months *vs.* NR, *P* < 0.001) and validation cohorts (*n* = 22: PFS, 1.6 months *vs.* 21.7 months, *P* = 0.004; OS, 3.0 months *vs.* 85.6 months)^18^.

A high tumor mutational burden (TMB) is a characteristic of dMMR/MSI-H tumors, and is known to include a higher number of neoantigens and responsiveness to immunotherapy. Chida and colleagues^17^ showed that a low TMB (< 10 mut/Mb) is associated with resistance to immunotherapy in GI patients, as evidenced by a lower ORR (0%) compared to 48.8% and a shorter PFS (2.3 months) compared to 25.6 months (*P* = 0.002)^17^. These findings are consistent with the results of a KEYNOTE 177 retrospective analysis^19^. In the current analysis, a low TMB (< 12 mut/Mb) was shown to be a predictor of resistance to ICIs in dMMR/MSI-H CRC, while a TMB greater than this threshold did not predict a response.

Currently, there is no definitive biomarker(s) that satisfactorily predicts resistance to ICIs in dMMR/MSI-H CRC. Further investigation into the molecular characteristics of dMMR/MSI-H tumors and the composition of the tumor microenvironment may provide more insight into the mechanism(s) underlying ICI resistance.

### Challenge of therapy after ICI resistance in dMMR/MSI-H mCRC

In addition to understanding the precise mechanism underlying ICI resistance, identifying effective treatments for ICI resistance is an urgent issue. Currently, there are two treatment strategies with limited published data in common use. The first strategy involves use of conventional chemotherapy with or without targeted therapy. The second strategy involves use of ICI in combination with other therapies, such as targeted therapy and chemotherapy or dual immunotherapy. A retrospective study showed that combining ICI with other treatments (e.g., anti-angiogenesis therapy in 15 patients and chemotherapy in 9 patients) resulted in better clinical outcomes compared to chemotherapy with or without targeted therapy for patients with MSI-H GI cancer who had progressed on ICIs^20^. Prospective studies have shown limited clinical benefit when combining anti-PD-L1 with TGF-β bifunctional fusion protein inhibitor (0% ORR) and anti-TIM3 (4.5% ORR) in patients with ICI-resistant MSI-H CRC^21,22^. Based on the results of CheckMate 142, there is reason to believe that combining anti-CTLA-4 with anti-PD-1 antibodies could potentially overcome ICI resistance in some patients. To further validate this hypothesis, a phase II trial is currently underway. The trial, known as CIRGC-01/CSWOG-C03 (NCT05426005), is determining the efficacy of cadonilimab, a PD-1/CTLA-4 bispecific antibody, in the treatment of advanced dMMR/MSI-H CRC refractory to PD-1/PD-L1 antibodies.

## ICI for locally advanced dMMR/MSI-H CRC

The purpose of neoadjuvant therapy in patients with CRC is to shrink tumors, reduce the shedding of tumor cells during surgery, eradicate micrometastases, and facilitate subsequent treatment decisions. The FOxTROT trial showed limited effectiveness of neoadjuvant chemotherapy for dMMR/MSI-H colon cancer, with only 3% (4/115) of patients achieving a pathologic CR (pCR) and 61% (70/115) of patients exhibiting no regression^23^. With the success of ICI treatment in patients with mCRC, efforts have been made to assess efficacy in the neoadjuvant setting.

NICHE was the first trial to be conducted involving neoadjuvant immunotherapy in localized dMMR/MSI-H colon cancer^24^. Specifically, 60% (12/20) of patients achieved a pCR and 95% of patients achieved a major pathologic response (≤ 10% of residual viable tumor) after receiving 2 doses of nivolumab and 1 dose of ipilimumab. A corollary trial (NICHE-2) also used the same treatment regimen^25^. A pCR was observed in 67% (72/107) of dMMR/MSI-H colon cancer patients. Among these patients, 64% had T4 tumors and 62% had N2 tumors.

Neoadjuvant therapy has a crucial role in the treatment of locally advanced rectal cancer (LARC) because of the specific anatomic location and function. The standard treatment for LARC involves neoadjuvant chemoradiation followed by surgery. In the case of dMMR LARC, a previous study revealed a CR rate of 30% (1 cCR + 8 pCR of 30 patients) when treated with chemoradiation^26^; however, a significant proportion of patients experience complications and AEs as a result of chemoradiation. These complications can affect bowel, urinary, and sexual function, as well as fertility. Furthermore, resection of the rectum and a permanent diverting colostomy can have a profound impact on quality of life.

Promising results have been observed in neoadjuvant immunotherapy for LARC in which all 12 stage II or III dMMR rectal cancer patients completed 6 months of dostarlimab treatment and achieved a cCR^27^. None of the patients had disease progression or recurrence during a median follow-up of 12 months^27^. Another phase II study conducted by Chen et al.^28^ reported that of 16 patients with T3/T4 or N+ dMMR rectal cancer who received 4 cycles of sintilimab, 75% (12/16) achieved a cCR or pCR. Furthermore, 9 patients opted for a “watch and wait” strategy and none of the patients experienced disease recurrence after a median follow-up of 17.2 months. Additionally, the PICC study demonstrated that locally advanced dMMR CRC patients who received toripalimab with or without celecoxib before surgery had a pCR rate of 76.5%^29^. These encouraging findings suggest that rectal cancer patients who achieve a cCR after ICI treatment may be suitable candidates for a “wait and watch” approach, thereby avoiding the need for surgery and potential complications associated with radiotherapy.

### Challenge of radiologic staging for locally advanced dMMR/MSI-H CRC

The accuracy of CT and MRI in T staging of CRC is considered unsatisfactory, especially for a T4b diagnosis. Previous reports have shown that CT has a sensitivity of 77% and a specificity of 70% in distinguishing between T3 and T4 in patients with colon cancer^30^, while MRI has an accuracy of 89.7% for T staging and 74.5% for N staging in patients with rectal cancer^31^. It is important to note that immune cell infiltration before or after immunotherapy, as well as the expansile growth pattern in dMMR/MSI-H CRC, may lead to overestimation of radiologic staging before ICI or underestimation after ICI. Only 1 of 29 patients with a pCR was correctly identified based on a radiologic response evaluation in the PICC trial^24^. To improve the accuracy of staging, a combination of endoscopy, CT/MRI, and even PET-CT may be necessary, as suggested by Cercek and colleagues^27^.

### Challenge of determining the ICI exposure time for locally advanced dMMR/MSI-H CRC

The appropriate exposure time to ICIs is a crucial factor that affects the CR rate. It has been reported that several months is required for a response to ICIs for patients with advanced dMMR/MSI-H tumors^32^; however, patients with locally advanced dMMR CRC have been shown to have a rapid therapeutic response to ICIs^24,25,27,28^. Neoadjuvant therapy involving 1 dose of ipilimumab and 2 doses of nivolumab resulted in pCR rates of 60% and 67% in dMMR colon cancer patients in the NICHE and NICHE-2 studies, respectively^24,25^. A near-cCR was achieved in patients who received 3 months of dostarlimab and all patients who received 6 months of dostarlimab achieved a cCR^27^. The PICC study demonstrated a pCR rate of 76.5% in patients receiving toripalimab or toripalimab plus celecoxib for 3 months^29^. In a study conducted by Chen and colleagues, the median time to reach a cCR was 5.2 months^28^. Therefore, based on the available evidence we suggest neoadjuvant immunotherapy as a means to avoid the need for surgery and chemoradiotherapy. A recommended course of treatment would involve 6 months of monotherapy. Alternatively, to reduce the size of the tumor for surgery 3 months of monotherapy or a combination of 1 dose of ipilimumab and 2 doses of nivolumab for neoadjuvant therapy and 6 months of overall perioperative duration may be sufficient. It is important to note that some patients may require a longer duration of neoadjuvant immunotherapy to achieve the desired treatment outcome.

### Challenge of selecting the appropriate locally advanced dMMR/MSI-H CRC patients for neoadjuvant immunotherapy

Neoadjuvant immunotherapy should be carefully prescribed to avoid patients missing the opportunity for surgery due to primary resistance to immunotherapy, inaccurate imaging staging, and severe immune-related AEs (irAEs). Generally, high-grade (≥ 3) irAEs occur in 10%–20% of patients treated with anti-PD-1/PD-L1 antibodies and in 40%–50% of patients treated with dual ICIs. While most irAEs occur within the first 6 months of treatment with a single-agent ICI, and even earlier with dual ICIs, the long-term implications of these toxicities should not be neglected, especially for patients treated in earlier stages. For locally advanced dMMR/MSI-H CRC patients, the incidence of high-grade irAEs or treatment-related AEs has been reported to be 0%–6% with neoadjuvant anti-PD-1 antibody compared to 13% with dual ICIs^24,27–29^. The NCCN guidelines suggest that neoadjuvant immunotherapy can be considered as a treatment option for patients with dMMR/MSI-H and T4b colon cancer, LARC (T3Nany, T1-2N1-2, T4Nany) and locally unresectable or medically inoperable CRC. Additionally, patients with Lynch syndrome, who are typically young, are considered suitable candidates for ICI treatment. Lynch syndrome is associated with an increased risk of metachronous CRC over time^33^; however, the risk is lower in patients who undergo subtotal colectomy compared to those who undergo segmental resection. Therefore, colectomy with ileorectal anastomosis is recommended for the treatment of patients with Lynch syndrome and CRC, although this procedure can significantly impact the quality of life. Previous studies have demonstrated that the effectiveness of immunotherapy is similar in Lynch syndrome and sporadic CRC^27^. Moreover, neoadjuvant immunotherapy may potentially eliminate the need for subtotal colectomy in young patients with Lynch syndrome.

Immunotherapy has undeniably brought significant benefits to dMMR/MSI-H CRC patients; however, there are several unresolved issues that need to be addressed. In the case of dMMR/MSI-H mCRC, the effectiveness of immunotherapy has been established, but there remains a need to explore the choice between single-agent or dual-ICI treatment, as well as primary or secondary resistance. The advantages of neoadjuvant immunotherapy are relatively clear, but there is ongoing controversy regarding the precise selection of patients, treatment duration, regimen selection, and efficacy evaluation. It is crucial that further studies be conducted and the results are published to provide more precise guidance for clinical practice.
